# The fraction of nitrous oxide in oxygen for facilitating lung collapse during one-lung ventilation with double lumen tube

**DOI:** 10.1186/s12871-020-01102-x

**Published:** 2020-07-22

**Authors:** Chao Liang, Yuechang Lv, Yu Shi, Jing Cang, Changhong Miao

**Affiliations:** 1 Department of Anesthesia, Zhongshan Hospital, Fudan University, Shanghai, China; 2 Department of Thoracic surgery, Zhongshan Hospital, Fudan University, Shanghai, China

**Keywords:** Nitrous oxide, Lung collapse, One-lung ventilation, Double lumen

## Abstract

**Background:**

The ideal fraction of nitrous oxide (N_2_O) in oxygen (O_2_) for rapid lung collapse remains unclear. Accordingly, this prospective trial aimed to determine the 50% effective concentration (EC_50_) and 95% effective concentration (EC_95_) of N_2_O in O_2_ for rapid lung collapse.

**Methods:**

This study included 38 consecutive patients undergoing video-assisted thoracoscopic surgery (VATS). The lung collapse score (LCS) of each patient during one-lung ventilation was evaluated by the same surgeon. The first patient received 30% N_2_O in O_2_, and the subsequent N_2_O fraction in O_2_ was determined by the LCS of the previous patient using the Dixon up-and-down method. The testing interval was set at 10%, and the lowest concentration was 10% (10, 20, 30, 40%, or 50%). The EC_50_ and EC_95_ of N_2_O in O_2_ for rapid lung collapse were analyzed using a probit test.

**Results:**

According to the up-and-down method, the N_2_O fraction in O_2_ at which all patients exhibited successful lung collapse was 50%. The EC_50_ and EC_95_ of N_2_O in O_2_ for rapid lung collapse were 27.7% (95% confidence interval 19.9–35.7%) and 48.7% (95% confidence interval 39.0–96.3%), respectively.

**Conclusions:**

In patients undergoing VATS, the EC_50_ and EC_95_ of N_2_O in O_2_ for rapid lung collapse were 27.7 and 48.7%, respectively.

**Trial registration:**

http://www.chictr.org/cn/ Identifier ChiCTR19 00021474, registered on 22 February 2019.

## Background

Rapid lung collapse facilitates intrathoracic surgical procedures, which are particularly important for minimally invasive video-assisted thoracoscopic surgery (VATS). It is well-known that when one-lung ventilation (OLV) begins, the nonventilated lung will undergo phase I lung collapse due to elastic recoil, which usually occurs within 60 s [1]. When phase I lung collapse ceases, presumably due to small airway closure, the slower phase II lung collapse begins, which mainly depends on continuous gaseous diffusion or absorption atelectasis. The previously recommended measures for hastening lung collapse include carbon dioxide insufflation of the pleural space [1] and intermittent airway suction [2]. However, to our knowledge, no studies have indicated that these measures actually achieve the intended result.

The rate of gas absorption in the nonventilated lung depends on the composition of the inspired gas [3, 4]. The oxygen (O_2_) fraction and solubility of any inert gas in the inspired mixture are important factors in the rate of gas absorption. If the inspired gas mixture contains a less soluble gas, such as nitrogen, the absorption rate is relatively slow and increases as O_2_ increases [4]. In contrast, when the inspired mixture contains a relatively soluble inert gas and O_2_, gas absorption is faster. In physiological terms, nitrous oxide (N_2_O) is highly soluble. In animal models [5, 6], it has been demonstrated that mechanical lung ventilation using an O_2_/N_2_O mixture will increase the rate of gaseous uptake from the non-ventilated lung and hasten its absorptive collapse. In addition, clinical studies have also indicated that, compared with an O_2_/air mixture or 100% O_2_, using an O_2_/N_2_O mixture before OLV prompts phase II lung collapse when a double-lumen endotracheal tube (DLT) or bronchial blocker (b-blocker) is used for lung isolation. Furthermore, this useful measure does not affect phase I lung collapse and cause hypoxia [7–9].

The commonly used N_2_O fraction in O_2_ for rapid lung collapse is 50% or 60% [6–8]; however, the proper fraction of N_2_O in O_2_ when this measure is used in thoracic procedures remains unclear. Accordingly, this prospective trial was designed to determine the 50% effective concentration (EC_50_) and 95% effective concentration (EC_95_) of N_2_O in O_2_ for rapid lung collapse.

## Methods

The present study was approved by the Institutional Review Board (IRB) of Zhongshan Hospital, Fudan University (Shanghai, China; IRB:B2018-314R), and written informed consent was obtained from all subjects who participated in the trial. The trial was registered before patient enrollment at http://www.chictr.org/cn/ (ChiCTR19 00021474, Principal investigator, Chao Liang, Date of registration, February 22, 2019). Patients scheduled to undergo elective VATS for lung cancer at the Zhongshan Hospital were enrolled in the present study. All patients underwent preoperative pulmonary function tests. Patients with evidence of bullae on chest radiography, abnormal expiratory recoil (forced expiratory volume in 1 s < 70% of predicted value), chronic obstructive pulmonary disease or severe asthma, major medical comorbidities, or anticipated pleural adhesion were excluded.

To avoid the potential effects of inhaled volatile anesthetic on oxygenation during OLV, all patients received total intravenous anesthesia. Propofol was administered using a target-controlled infusion (TCI) device (Cardinal Health, Basingstoke, United Kingdom) based on a three-compartment population pharmacokinetic model defined by Schnider et al. [10]. Anesthesia was induced using propofol TCI (target plasma concentration set at 4.0 μg ml^− 1^), remifentanil (0.2 μg kg^− 1^ min^− 1^), fentanyl 1 μg kg^− 1^, and rocuronium bromide 0.6 mg kg^− 1^. Anesthesia was maintained using propofol TCI (target plasma concentration set at 3.0 μg ml^− 1^) infusion and intermittent boluses rocuronium. Tidal volumes were 8 mL kg^− 1^ ideal body weight during both two-lung ventilation (2LV) and OLV without positive end-expiratory pressure (PEEP). The 100% O_2_ was introduced by a mask during induction for 3 min. Patients were intubated using an appropriate-size, left-sided, DLT; the position of the DLT was confirmed using fiberoptic bronchoscopy (FOB). The selected N_2_O/O_2_ admixture was then introduced and continued during positive pressure ventilation until the start of OLV. The patients were placed in the lateral position, and the position of the DLT was reconfirmed and adjusted using FOB as needed. At the time of skin incision, the DLT lumens were opened to the atmosphere for 60 s, then the nonventilated lumen of the DLT was clamped for gas uptake, and OLV of the dependent lung was started with a fraction of inspired oxygen of 1.0.

### Measurement

Given that all procedures were conducted using VATS, lung collapse was scored via video view. Surgeons were blinded to the gas composition, assessing LCS at 5 min after pleural opening using a verbal rating scale [7] scored from 0 (no lung deflation) to 10 (maximal lung collapse). FOB was used to diagnose and correct the problem when lung isolation was unsatisfactory. Baseline arterial blood gas of each patient was obtained preoperatively while patients breathed room air. After anesthesia induction, the right or left radial artery was cannulated, and blood gas samples were analyzed every 10 min for the first 30 min of OLV. The lowest O_2_ saturation (SpO_2_) during OLV and the time required to open the lung pleura (time from start of OLV until pleural opening), end-tidal carbon dioxide, heart rate, and arterial blood pressure were also recorded. End-tidal O_2_ or N_2_O was recorded every minute from the start of OLV using an anesthetic analyzer that was a component of the anesthesia machine (IntelliVue G5, Phillips, Andover, MA, USA).

To calculate the EC_50_ and EC_95_ of N_2_O in O_2_, the N_2_O fraction in O_2_ in the first case was 30%, and the subsequent N_2_O fraction was determined by the LCS of the previous patient using the Dixon up-and-down method. The testing interval was set at 10%, and the lowest concentration of N_2_O was 10%. “Successful lung collapse” was defined as an LCS ≥ 8, and the N_2_O fraction in the subsequent patient was decreased by 10%. An LCS < 8 was regarded as “fail”, and the N_2_O fraction in the subsequent patient was increased by 10%.

### Statistical analysis

Statistical analysis was performed using SPSS version 19.0 (IBM Corporation, Armonk, NY, USA) and Excel 2007 (Microsoft Corporation, Redmond, WA, USA). Patient characteristics were expressed as mean and standard deviation (SD) or number. Continuous variables were analyzed using the t-test and categorical variables were analyzed using the chi-squared test. The mean of the mid-point of all fail/success pairs was used to calculate N_2_O EC_50_ using up-and-down method described by Dixon and Massey, and a minimum of 8 crossover pairs were required for the analysis [11]. A dose-response curve was determined using probit analysis and interpolation was performed to obtain EC_50_ and EC_95_ with 95% corresponding confidence interval (CI).

## Results

**Fig. 1:**
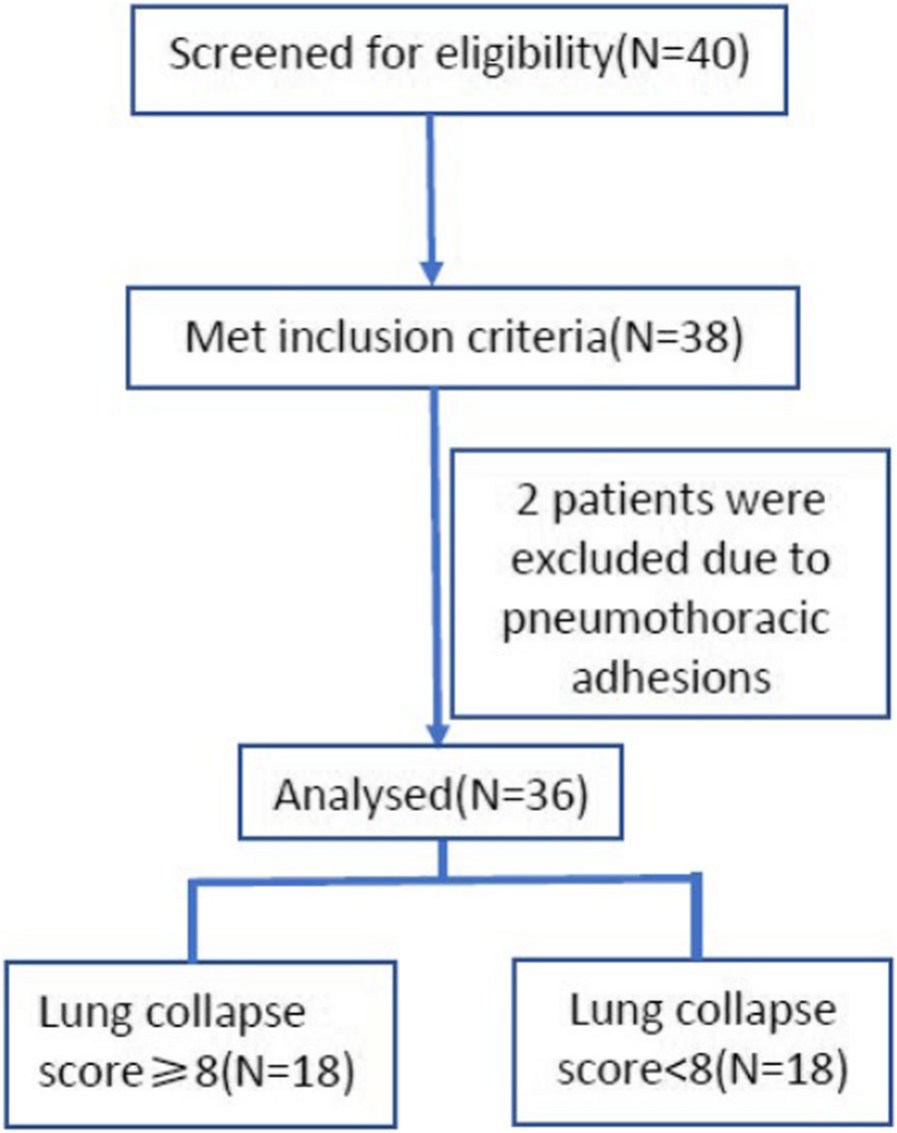
Flow diagram of participants

The eligibility of 40 patients was assessed and 38 were recruited for the study (Fig. 1). All patients had satisfactory lung isolation and did not require correction of DLT malpositioning or discontinuation of OLV. An additional two patients were excluded from the study due to pneumothoracic adhesions and, consequently, difficult assessment of LCS. Ultimately, therefore, 36 patients were analyzed. The demographic characteristics of the patients are summarized in Table 1.

**Table 1:** Demographic data of study population

American Society of Anesthesiologists score	2 (1–3)
**Age(y)**	**52.5 ± 9.2**
**Gender (male/female)**	**19/17**
**Weight (kg)**	**57.6 ± 9.2**
**Height (cm)**	**157 ± 8.6**
**Smokers/Non-smokers**	**3/33**
^**a**^**Pleural opening(s)**	**59.6 ± 12.2**
**FEV1 (% of predicted)**	**84.6 ± 12.2**
**FVC (% of predicted)**	**82.3 ± 9.2**
**FEV1/FVC**	**80.2 ± 8.8**
**Surgery type**	
**VATS-R**	**20**
**VATS-L**	**16**

FEV1 = forced expiratory volume at 1 s; FVC = forced

vital capacity

^a^Time from incision to pleural opening

VATS = video-assisted thoracoscopic surgery

**Fig. 2:**
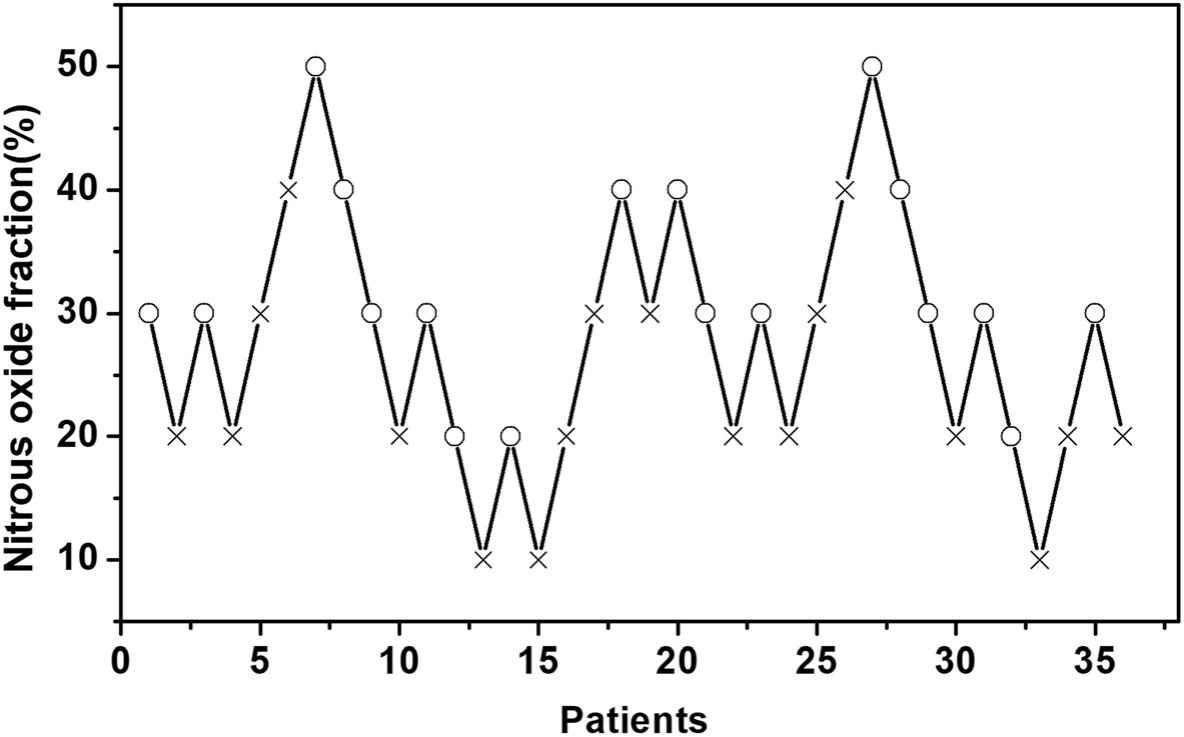
The sequential lung collapse score of 36 patients to nitrous oxide with the up-and-down method. × = lung collapse score < 8; ○ = lung collapse score ≥ 8

**Fig. 3:**
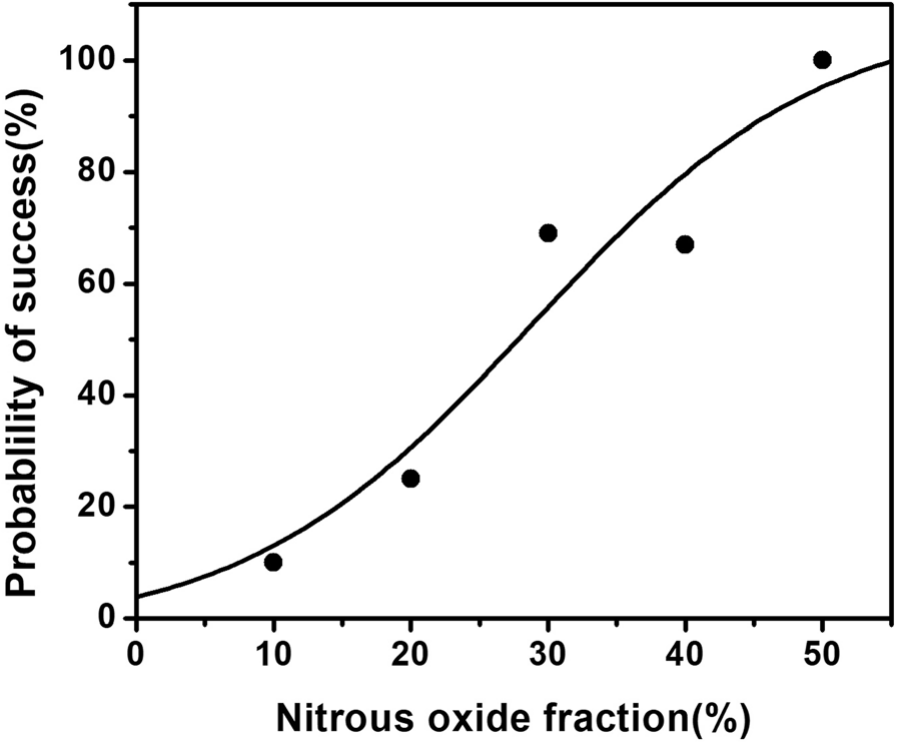
Dose-response curve for nitrous oxide plotted using probit analysis. The 50% effective concentration was 27.7% (95% confidence interval, 19.9–35.7%). The 95% effective concentration was 48.7% (95% confidence interval, 39.0–96.3%)

The N_2_O fraction success data of LCS for patients obtained using the up-and-down method are presented in Fig. 2. This was further analyzed by probit regression analysis. The EC_50_ of N_2_O in O_2_ for rapid lung collapse was 27.7% (95% confidence interval [CI] 19.9–35.7%). The EC_95_ of N_2_O in O_2_ for rapid lung collapse was 48.7% (95% CI 39.0–96.3%). The N_2_O fraction in O_2_ and percentages of patients who achieved successful lung collapse (i.e., LCS ≥ 8) are summarized in Table 2. The fraction-success curve of N_2_O plotted from probit analysis of individual N_2_O fractions and the respective LCS is presented in Fig. 3. Clinically significant desaturation (SpO_2_ < 90%) requiring alveolar recruitment maneuvers or other interventions did not occur in any patient. During the investigation period, no other intraoperative hemodynamic events (hypotension, tachycardia, and bradycardia) were recorded or required intervention.

**Table 2:** Percentages of patients who had successful lung collapse score (lung collapse score equal to or more than 8).

Nitrous oxide fraction in each subgroup (%)	Success rate
**10**	**0% (0/3)**
**20**	**25% (3/12)**
**30**	**68% (9/13)**
**40**	**67% (4/6)**
**50**	**100% (2/2)**

## Discussion

The use of an N_2_O/O_2_ mixture is a useful method for rapid lung collapse. The present study determined that the EC_50_ of N_2_O in O_2_ for rapid lung collapse was 27.7%.

The underlying mechanism of an N_2_O/O_2_ inspired gas mixture leads to rapid lung collapse may attributed to a “second gas” effect, which is the rapid absorption of N_2_O facilitating O_2_ uptake, or to a concentration effect, or to gas solubility [12]. During OLV, the nonventilated lung collapses initially due to elastic recoil, and the remaining gas is then removed by absorption into the pulmonary capillary blood [6]. Thus, in the present study, for complete lung collapse by elastic recoil, both nonventilated and ventilated lumens of the DLT were opened to the atmosphere for 60 s, then the nonventilated lumen was clamped for gas uptake. The average time of plural opening in the present study was approximately 60 s (mean, 59.6 ± 12.2 s), which is consistent with previous studies reporting on plural opening in VATS [7]. Then, a verbal rating scale [7, 8], scored from 0 (no lung deflation) to 10 (maximal lung collapse), was used by the surgeon to score the patient’s lung collapse condition. Other studies [13, 14] have also used a four-point ordinal scale (1, extremely poor to no collapse of the lung; 2, poor partial collapse with interference with surgical exposure; 3, good total collapse, but the lung still contained residual air; and 4, excellent to complete collapse with perfect surgical exposure). To evaluate the condition of the lung, however, defining a “success” and “fail” condition is a necessary step for determining EC_50_ using the up-and-down method. Compared with a four-point ordinal scale, a verbal rating scale from 0 to 10 appears to be more accurate for scoring lung collapse condition. Moreover, in our pilot study, virtually all surgeons regarded LCS ≥ 8 as a proper condition for lung manipulations; thus, we defined LCS ≥ 8 as “success” and < 8 as “fail”.

In a study investigating the use of a b-blocker as a lung isolation tool, the LCS of 50% N_2_O in O_2_ was significantly higher compared with that of 100% O_2_ at 5 min after opening the pleura; however, < 50% patients’ LCS was ≥8 [7]. In another study, in which DLT was used as the lung isolation tool, when 50% N_2_O was applied, the average LCS was 9 at 10 min after opening the pleura, although the investigators did not report LCS at 5 min after opening the pleura [8]. When 30% N_2_O in O_2_ was used in our pilot study, approximately 50% of patients had an LCS ≥ 8. Differences in LCS 5 min after opening the pleura between our study and the study investigating b-blockers as the lung isolation tool may largely be attributed to the different isolation tools and the surgeon’s personal LCS scoring criteria.

In previous studies [7, 8], the target gas mixtures of N_2_O and O_2_ were used at the time of preoxygenation during anesthesia induction, and the gas concentrations before OLV were equal to the target concentrations. In the present study, 100% O_2_ was used for preoxygenation, and the selected N_2_O and O_2_ gas mixtures were then used after intubation. However, before OLV, all selected N_2_O and O_2_ gas mixtures were equal to the target mixtures. Therefore, it appears that using O_2_ for induction, and switching to N_2_O and O_2_ after intubation is more applicable because a “more O_2_ induction period” is safer than one that involves less. Regarding operation type, all patients in the present study underwent VATS for lung surgery, which is the primary surgery type for lung tumors, and the enrolled cases in previous studies mainly underwent open thoracotomies. Compared with open thoracotomies, the lung collapse condition is more important for VATS; thus, data from the present study are more applicable to modern clinical practice(s).

The present study had several limitations. First, for the purposes of this study, we determined the success or failure of lung collapse based on the surgeons’ scoring scale, which was not entirely objective. However, similar to the methods used in previous studies, using more objective criteria, such as the distance of the collapsed lung to the chest wall, appears to be less clinically relevant due to varying sizes of patient chests. Therefore, the most clinically relevant assessment of the lung collapse condition is the surgeon’s opinion. Second, the tidal volumes were 8 mL kg^− 1^ ideal body weight during both 2LV and OLV without PEEP. However, this has been associated with increased postoperative complications and mortality [15]. Furthermore, an adequate amount of PEEP was shown to be effective in reducing stress to the dependent lung and V/Q mismatch [16]. Applying PEEP to the dependent lung should also influence the primary outcome. In fact, LCS was assessed by a surgeon who could have been confounded by a more inflated dependent lung. Third, all patients in the present study demonstrated relatively normal results on pulmonary function testing (including 3 smokers) and body mass indices. As such, the results of our study may not be applicable to patients with poor pulmonary function test results, or to obese patients and/or smokers. Lastly, the duration of administration of the O_2_/N_2_O admixture was from the confirmation of DLT with FOB to the time of skin incision, and unfortunately, we did not record the time of this period. These concerns may be addressed in future studies.

## Conclusion

When a DLT was used for lung isolation in patients undergoing VATS, the EC_50_ and EC_95_ of N_2_O in O_2_ during 2LV for accelerating lung collapse during OLV were 27.7 and 48.7%, respectively.

## Data Availability

Reasonable requests for access to the datasets used and/or analysed during this study can be made to the corresponding author.

## Abbreviations

N_2_O: Nitrous oxide
O_2_: Oxygen
EC_50_: 50% effective concentration
EC_95_: 95% effective concentration
VATS: Video-assisted thoracoscopic surgery
LCS: Lung collapse score
OLV: One-lung ventilation
DLT: Double-lumen tube
b-blocker: Bronchial blocker
FEV_1_: Forced expiratory volume at 1 s
TCI: Target-controlled infusion
FOB: Fiberoptic bronchoscopy
CI: Confidence intervals

## References

[1] Landreneau RJ, Mack MJ, Hazelrigg SR, Dowling RD, Acuff TE, Magee MJ, Ferson PF (1992). Video-assisted thoracic surgery: basic technical concepts and intercostal approach strategies. Ann Thorac Surg.

[2] Baraka A (1998). Hazards of carbon dioxide insufflation during thoracoscopy. Br J Anaesth.

[3] Dale WA, Rahn H (1952). Rate of gas absorption during atelectasis. Am J Phys.

[4] Joyce CJ, Baker AB, Kennedy RR: **Gas uptake from an unventilated area of lung: computer model of absorption atelectasis**. *J Appl Physiol (1985)* 1993, **74**(3):1107–1116. 10.1152/jappl.1993.74.3.1107 8482648

[5] Joyce CJ, Baker AB, Parkinson R, Zacharias M (1996). Nitrous oxide and the rate of gas uptake from an unventilated lung in dogs. Br J Anaesth.

[6] Pfitzner J, Peacock MJ, Pfitzner L (2001). Speed of collapse of the non-ventilated lung during one-lung anaesthesia: the effects of the use of nitrous oxide in sheep. Anaesthesia.

[7] Yoshimura T, Ueda K, Kakinuma A, Sawai J, Nakata Y (2014). Bronchial blocker lung collapse technique: nitrous oxide for facilitating lung collapse during one-lung ventilation with a bronchial blocker. Anesth Analg.

[8] Ko R, McRae K, Darling G, Waddell TK, McGlade D, Cheung K, Katz J, Slinger P (2009). The use of air in the inspired gas mixture during two-lung ventilation delays lung collapse during one-lung ventilation. Anesth Analg.

[9] Joyce CJ, Baker AB (1995). What is the role of absorption atelectasis in the genesis of perioperative pulmonary collapse?. Anaesth Intensive Care.

[10] Schnider TW, Minto CF, Gambus PL, Andresen C, Goodale DB, Shafer SL, Youngs EJ (1998). The influence of method of administration and covariates on the pharmacokinetics of propofol in adult volunteers. Anesthesiology.

[11] Dixon WJ (1991). Staircase bioassay: the up-and-down method. Neurosci Biobehav Rev.

[12] Pfitzner J, Peacock MJ, Harris RJ (2001). Speed of collapse of the non-ventilated lung during single-lung ventilation for thoracoscopic surgery: the effect of transient increases in pleural pressure on the venting of gas from the non-ventilated lung. Anaesthesia.

[13] El-Tahan MR (2015). A comparison of the disconnection technique with continuous bronchial suction for lung deflation when using the Arndt endobronchial blocker during video-assisted thoracoscopy: a randomised trial. Eur J Anaesthesiol.

[14] Li Q, Zhang X, Wu J, Xu M (2017). Two-minute disconnection technique with a double-lumen tube to speed the collapse of the non-ventilated lung for one-lung ventilation in thoracoscopic surgery. BMC Anesthesiol.

[15] Blank RS, Colquhoun DA, Durieux ME, Kozower BD, McMurry TL, Bender SP, Naik BI (2016). Management of one-lung Ventilation: impact of tidal volume on complications after thoracic surgery. Anesthesiology.

[16] Spadaro S, Grasso S, Karbing DS, Fogagnolo A, Contoli M, Bollini G, Ragazzi R, Cinnella G, Verri M, Cavallesco NG (2018). Physiologic evaluation of ventilation perfusion mismatch and respiratory mechanics at different positive end-expiratory pressure in patients undergoing protective one-lung ventilation. Anesthesiology.

